# Heterogeneity of CD34 and CD38 expression in acute B lymphoblastic leukemia cells is reversible and not hierarchically organized

**DOI:** 10.1186/s13045-016-0310-1

**Published:** 2016-09-22

**Authors:** Zhiwu Jiang, Manman Deng, Xinru Wei, Wei Ye, Yiren Xiao, Simiao Lin, Suna Wang, Baiheng Li, Xin Liu, Gong Zhang, Peilong Lai, Jianyu Weng, Donghai Wu, Haijia Chen, Wei Wei, Yuguo Ma, Yangqiu Li, Pentao Liu, Xin Du, Duanqing Pei, Yao Yao, Bing Xu, Peng Li

**Affiliations:** 1 State Key Laboratory of Respiratory Disease, Guangzhou Institutes of Biomedicine and Health, Chinese Academy of Sciences, 190 Kaiyuan Avenue, Science Park, Guangzhou, Guangdong 510530 China; 2 Key Laboratory of Regenerative Biology, South China Institute for Stem Cell Biology and Regenerative Medicine, Guangzhou Institutes of Biomedicine and Health, Chinese Academy of Sciences, Guangzhou, 510530 China; 3 Guangdong Provincial Key Laboratory of Stem Cell and Regenerative Medicine, South China Institute for Stem Cell Biology and Regenerative Medicine, Guangzhou Institutes of Biomedicine and Health, Chinese Academy of Sciences, Guangzhou, 510530 China; 4 Department of Hematology, The First Affiliated Hospital of Xiamen University, Xiamen, 361003 China; 5 Department of Hematology, Nanfang Hospital, Southern Medical University, Guangzhou, 510515 China; 6 Shenzhen Institutes of Advanced Technology, Chinese Academy of Sciences, 1068 Xueyuan Avenue, Shenzhen University Town, Shenzhen, 518055 China; 7 Key Laboratory of Functional Protein Research of Guangdong Higher Education Institutes, Institute of Life and Health Engineering, College of Life Science and Technology, Jinan University, Guangzhou, 510632 China; 8 Department of Hematology, Guangdong Provincial People’s Hospital, Guangzhou, 510500 China; 9 Guangzhou SALIAI Stem Cell Science and Technology Co. Ltd, Guangzhou, 510000 China; 10 Guangdong Cord Blood Bank, Guangzhou, 510000 China; 11 Yikang Tailai Technology Co. Ltd, Guangzhou, 510530 China; 12 Department of Hematology, Medical College, Jinan University, Guangzhou, 510632 China; 13 Key Laboratory for Regenerative Medicine of Ministry of Education, Jinan University, Guangzhou, 510632 China; 14 Wellcome Trust Sanger Institute, Hinxton, Cambridge, CB10 1HH England UK; 15 Drug Discovery Pipeline, Guangzhou Institutes of Biomedicine and Health, Chinese Academy of Sciences, Guangzhou, 510530, China

**Keywords:** B-ALL, Leukemia stem cell, Heterogeneity, Xenografts

## Abstract

The existence and identification of leukemia-initiating cells in adult acute B lymphoblastic leukemia (B-ALL) remain controversial. We examined whether adult B-ALL is hierarchically organized into phenotypically distinct subpopulations of leukemogenic and non-leukemogenic cells or whether most B-ALL cells retain leukemogenic capacity, irrespective of their immunophenotype profiles. Our results suggest that adult B-ALL follows the stochastic stem cell model and that the expression of CD34 and CD38 in B-ALL is reversibly and not hierarchically organized.

**Electronic supplementary material:**

The online version of this article (doi:10.1186/s13045-016-0310-1) contains supplementary material, which is available to authorized users.

Currently, the long-term survival of adult B-ALL patients is less than 50 % [1–4]. To improve the cure and survival rates of adults, there is an increasing need to understand the biology of B-ALL and to characterize the leukemia-initiating cells (LICs) in B-ALL if they exist [5, 6]. Primary B-ALL cells from 25 adult patients (Additional file 1: Table S1) were intravenously transplanted into groups of adult NSI mice [7–9] that had undergone preconditioning total body irradiation. Twelve of the 25 samples engrafted successfully (Additional file 2: Table S2). In the 12 cases of successful engraftment, the mice died or developed severe clinical signs suggestive of leukemia and requiring euthanasia (Additional file 3: Table S3). Consistent with primary xenografts, the human B-ALL cells that expressed CD19, CD34, CD38, and CD45 in serial transplanted NSI mice closely recapitulated the immunophenotypes of the original patient (Additional file 4: Figure S1, S2A). The morphology of leukemic cells in the peripheral blood, spleens, and bone marrow (BM) of xenografts resemble the original patient samples (Additional file 5: Figure S2B). The CD34 and CD38 expression profiles of engrafted B-ALL cells from transplanted NSI mice resemble the original patient samples (Additional file 5: Figure S2A and Additional file 6: Figure S3).

CD34 and CD38 molecules had been used as surface markers to distinguish LICs [10, 11]. To identify whether CD34 and CD38 can be used as LICs markers in B-ALL cells, we purified CD34^+^CD38^−^, CD34^+^CD38^+^, and CD34^−^CD38^+^ fractions from the xenografts of patients #1 and #3. We subsequently performed limited dilution transplantation of these subpopulations in NSI mice. The purities of the subpopulations were 97.3 % ± 0.89 (*n* = 12, Additional file 7: Figure S4). The xenotransplantation results showed that each fraction of B-ALL cells from xenografts of patients #1 and #3 was capable of engrafting in NSI mice (Additional file 3: Table S3). Each subpopulation from xenografts of patients individually reconstituted B-ALL that contained CD34^+^CD38^−^, CD34^+^CD38^+^, and CD34^+^CD38^−^ fractions in NSI mice (Fig. 1). Genome-wide expression profile analysis revealed that each population was clustered closely in patients #1 and #3 (Additional file 8: Figure S5). RNA-Seq results were further validated by measuring the messenger RNA (mRNA) levels of oncogenesis-related genes using quantitative RT-PCR (Additional file 9: Figure S6).

**Fig. 1:**
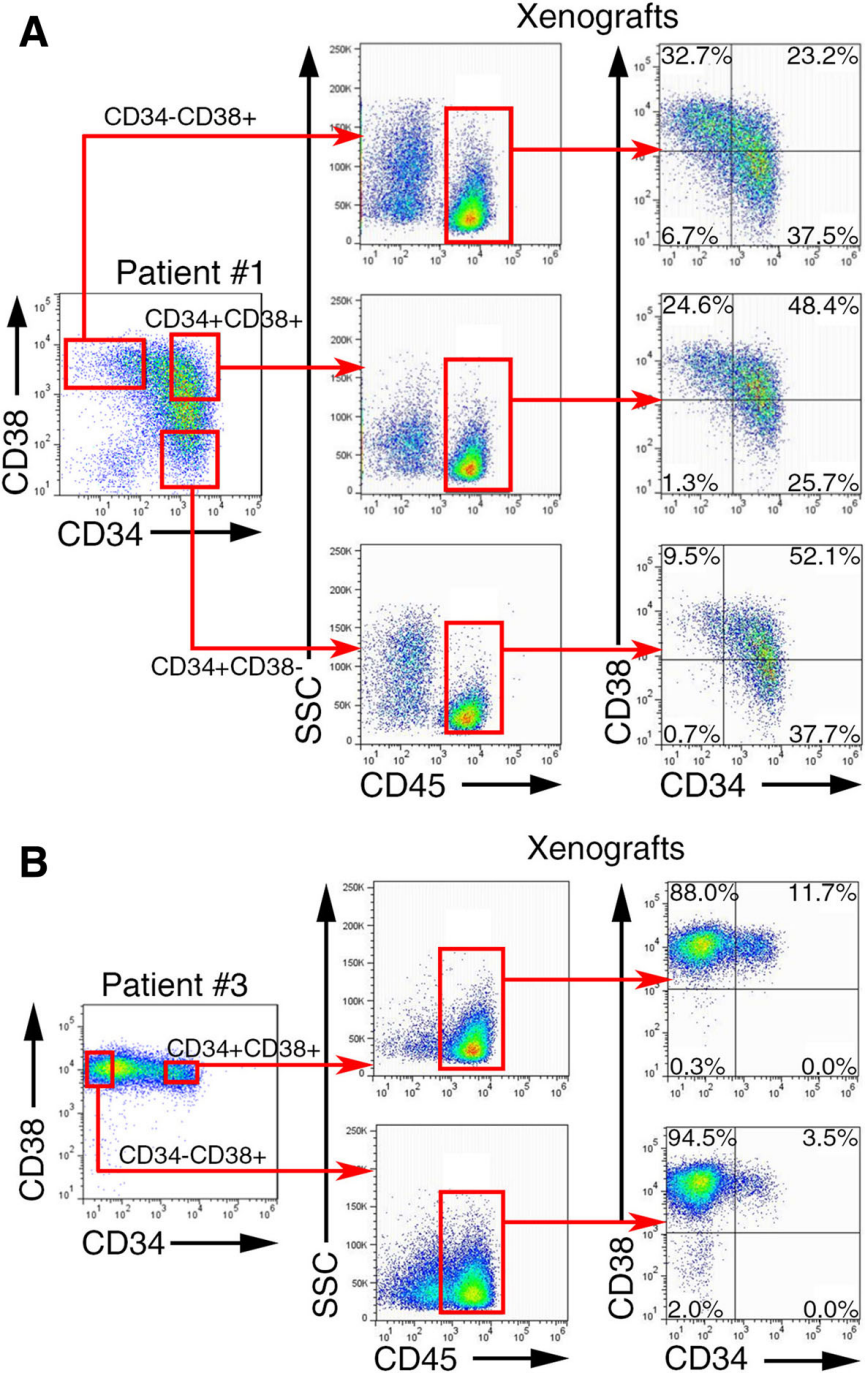
Subpopulations of adult B-ALL cells reconstituted the leukemia in xenografts. Subpopulations of CD34^+^CD38^−^, CD34^+^CD38^+^, and CD34^−^CD38^+^ from xenografts of patients #1 and #3 were purified and injected into groups of NSI mice. **a** Representative FACS analysis of gated hCD45^+^ BM cells from NSI recipients that were transferred with different subpopulations of engrafted B-ALL cells from patient #1. **b** Representative FACS analysis of gated hCD45^+^ BM cells from NSI mice that were transferred with CD34^+^CD38^+^ and CD34^−^CD38^+^ fractions of engrafted B-ALL cells from patient #3

Next, we investigated whether expanded B-ALL cells in vitro still maintain original expression profiles of CD34 and CD38 and the LIC capacity. B-ALL cells from 11 of the 12 patient samples that successfully engrafted in NSI mice attached to OP9 cells and proliferated vigorously for at least 2 months (Additional file 10: Table S4). We then monitored the expression profiles of CD34 and CD38 in B-ALL cells in differential time. To our surprise, CD34^+^CD38^−^ and CD34^+^CD38^+^ subpopulations from patient #1 disappeared gradually in culture (Fig. 2a). Six weeks after co-culture with OP9 cells, all remaining leukemic cells were CD34^−^CD38^+^ (Additional file 10: Table S4). To investigate whether CD34^−^CD38^+^ B-ALL cells after culture were still capable of engrafting in mice, we further purified cultured CD34^−^CD38^+^ B-ALL cells from patients #1, #4, and #7 and injected them into groups of NSI mice. After 4 weeks transplantation, cultured CD34^−^CD38^+^ B-ALL cells from patient reconstituted B-ALL consisting of CD34^+^CD38^−^, CD34^+^CD38^+^, and CD34^−^CD38^+^subpopulations in mice (Fig. 2b and Additional file 11: Table S5). Whole exome-sequencing analysis [12] showed that B-ALL cells from co-culture and B-ALL cells from xenografts shared similar SNP profiles (Additional file 12: Figure S7). This result indicates B-ALL cells maintain stable genetic characteristics irrespective of phenotypes. Our results also showed that individual B-ALL cells successfully engrafted in 4 of the 70 hosts and repopulated original surface profiles (Additional file 13: Figure S8 and Additional file 14: Table S6, detailed  methodological information was included in Additional file 17: supplementary methods.).

**Fig. 2:**
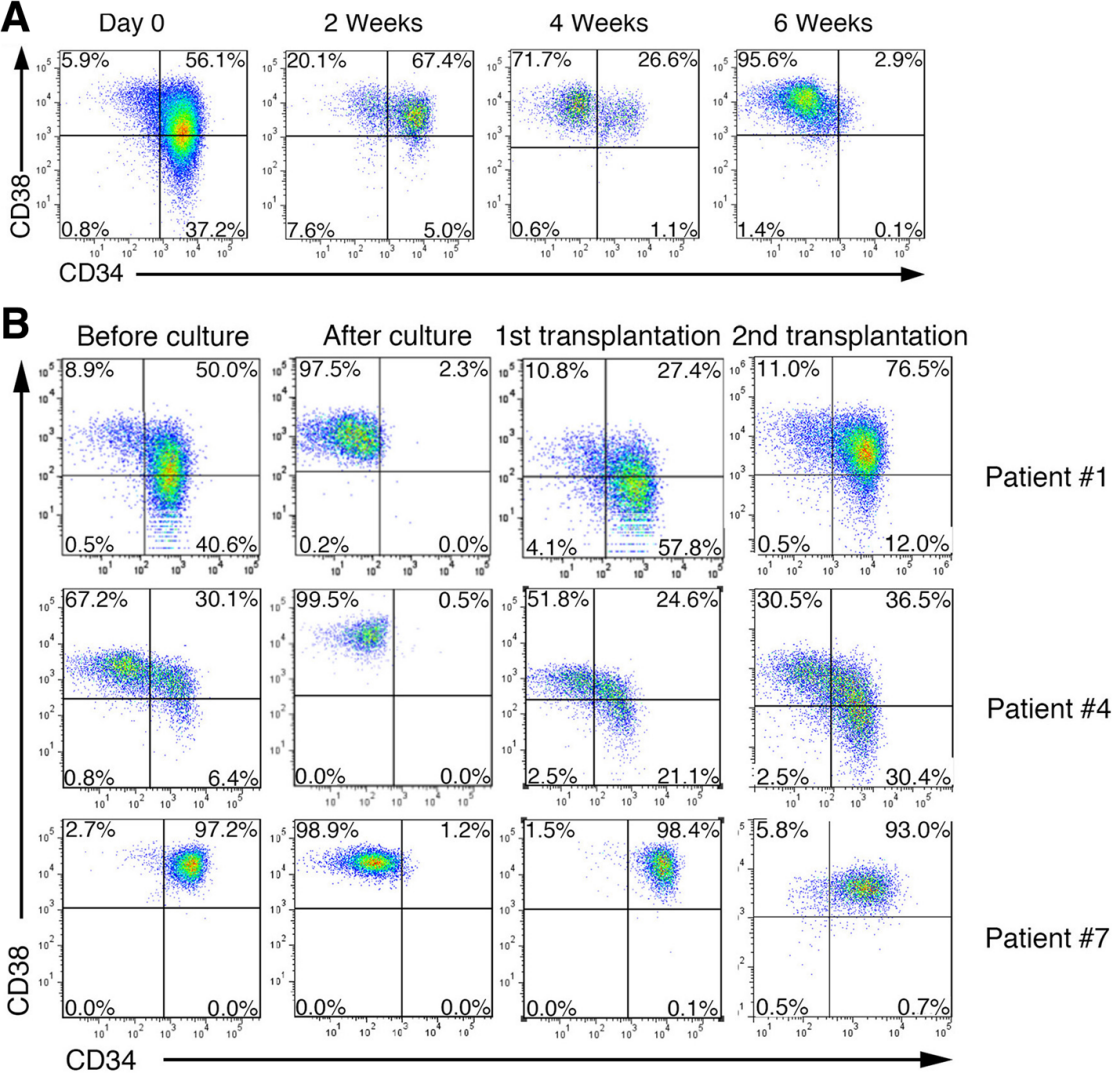
Cultured leukemic cells maintain the stem cell capacity. **a** Representative FACS analysis of CD34 and CD38 expression profiles in primary B-ALL cells from patient #1 in OP9 co-culture at indicated time points. **b** B-ALL cells from xenografts of patients #1, #4, and #7 were co-cultured with OP9 stromal cells. After 6 weeks, cultured B-ALL cells were subjected to FACS analysis. Then CD34^−^CD38^+^ populations were enriched from cultured B-ALL cells and were subsequently injected into groups of NSI mice for serial transplantations. Eight weeks after transplantation, BM cells from xenografts were subjected for FACS analysis. Representative FACS analysis of gated CD45^+^ cells from xenografts or co-cultures

In conclusion, our results demonstrate that leukemic blasts, irrespective of CD34 and CD38 expression, are able to engraft immunodeficient mice and reconstitute the original leukemia. Furthermore, we provide evidence that the heterogeneity of CD34 and CD38 expression in B-ALL obtained from patients reverses in different microenvironments. This phenotypic plasticity contrasts the cancer stem cell model, which largely attributes heterogeneity to irreversible epigenetic changes.

## Additional files

Additional file 1: Table S1. Characteristics of the 25 patients. (DOCX 17 kb) Additional file 2: Table S2. Clinical information of the 25 B-ALL patients. (DOCX 25 kb) Additional file 3: Table S3. Summary of mice engrafted with different subpopulations of primary B-ALL cells from xenografts. (DOCX 31 kb) Additional file 4: Figure S1. Reconstitution of adult B-ALL in NSI mice. (A) Left, spleens from of an NSI mouse engrafted with B-ALL cells and an NSI mouse not injected with B-ALL cells. Right, weights of the spleens from the mice engrafted with or without B-ALL (0.69 ± 0.24 versus 0.02 ± 0.02; *p* = 0.001). Data are shown as mean ± SEM. (B) Representative FACS analysis of primary leukemic cells from patient #1, BM cells from xenografts of patient #1 (mouse #140815N1), and BM cells from a healthy donor. Red lines highlight the median values of hCD45 expression levels in leukemic cells and in lymphoid cells. (C) Representative FACS analysis of hCD10 and CD19 cells from patient #1 and leukemic cells from xenograft of patient #1 (mouse #140815N1). (D) Representative FACS analysis of hCD14 and hCD33 cells from BM cells from xenograft of patient #1 (mouse #140815N1). (JPG 648 kb) Additional file 5: Figure S2. Immunophenotypes of the leukemic cells remain stable for serial transplantations. (A) Representative FACS analysis of leukemic cells from patients #1, #3, and #7 and leukemic cells from their xenografts after serial transplantations. (B) Representative of H&E staining of blood smear (peripheral blood), spleen and bone marrow of xenografts of patient #1 and patient #1 BM. Scale bars represent 100 μm (JPG 1500 kb) Additional file 6: Figure S3. Characterizations of CD34 and CD38 expression profiles in B-ALL cells in patients and xenografts. BM cells of xenografts were analyzed by FACS to determine their expression profiles of CD34 and CD38. (JPG 1305 kb) Additional file 7: Figure S4. Reevaluation of purities of primary B-ALL cells after sorting. (A) Representative FACS analysis of sorted CD34^+^CD38^−^, CD34^+^CD38^+^, and CD34^−^CD38^+^ fractions from xenograft of patient #1. (B) Representative FACS analysis of purified CD34^+^CD38^+^ and CD34^−^CD38^+^ fractions from xenograft of patient #3. (JPG 550 kb) Additional file 8: Figure S5. Gene expression pattern in subpopulations of B-ALL cells. (A, B) The immunophenotypes of patients #1 and #3 respected to CD34 and CD38 expression for RNA-Seq analysis. (C) The expression levels of CD10, CD20, CD34, CD19, CD38, and CD33 in indicated subpopulations from patients #1, #3, and two healthy donors. (D) Hierarchical clustering shows different subpopulations of patient #1 and #3 are grouped together. (JPG 758 kb) Additional file 9: Figure S6. The expression level of genes was confirmed by qRT-PCR. (A) Relative expression levels of indicated genes in patient #1 (blue) and patient #3 (red) cells were measured by qRT-PCR. The results were normalized to β-ACTIN mRNA levels and represent the means ± SEM. (*n* = 3). The qRT-PCR results were compared to the expression levels of these genes in B-ALL cells from patient #1 and patient #3 indicated by RNA-Seq analysis. (JPG 516 kb) Additional file 10: Table S4. Characteristics of leukemic cells in co-culture with OP9 stromal cells. (DOCX 18 kb) Additional file 11: Table S5. Engrafted mice transplanted with cultured leukemic cells. (DOCX 19 kb) Additional file 12: Figure S7. Leukemic cells’ SNPs are conserved in ex vivo. (A) Whole exome sequencing of the leukemia in xenografts (before culture, B. C.), after culture (A. C.) and after transplantation (A. T.) revealed the presence of genome mutations. Similar rates of nonsynonymous and synonymous single nucleotide variants (SNVs) within the leukemia of patient #1 and patient #7. (B, C) Venn diagram present B-ALL cells from co-culture and B-ALL cells from xenografts share similar nonsynonymous SNP profiles. (JPG 1513 kb) Additional file 13: Figure S8. Single cell assays of primary adult B-ALL cells. (A) Each NSI mouse was injected with a single primary B-ALL cell. Sixteen weeks after transplantation, the BM compartments of hosts were subjected to FACS analysis. (B) Representative FACS analysis of B-ALL cells used for single cell transplantation (Before, left) and gated hCD45^+^ cells from the BM compartments of the successfully grafted hosts (After, right). (JPG 494 kb) Additional file 14: Table S6. Summary of single B-ALL cell xenotransplantation. (DOCX 27 kb) Additional file 15: Table S7. Antibodies used for multicolor FACS analysis and cell sorting. (DOCX 19 kb) Additional file 16: Table S8. Primers used for qRT-PCR and PCR. (DOCX 20 kb) Additional file 17: Supplementary methods (Additional file 15: Table S7 and Additional file 16: Table S8). (DOCX 44 kb)

## Abbreviations

B-ALL: Acute B lymphoblastic leukemia
BM: Bone marrow
LICs: Leukemia-initiating cells
NSI: NOD/SCID/IL2^−/−^
