# Supplementary material for: Heterogeneity of CD34 and CD38 expression in acute B lymphoblastic leukemia cells is reversible and not hierarchically organized
Source: J Hematol Oncol. 2016 Sep 22;9:94. doi: 10.1186/s13045-016-0310-1 (PMC5034590; doi:10.1186/s13045-016-0310-1)
Supplement: Additional file 1: Table S1. — Characteristics of the 25 patients. (DOCX 17 kb) [file 13045_2016_310_MOESM1_ESM.docx]

**Table S1. Characteristics of the 25 patients**

| Characteristics | Value |
| --- | --- |
| Race | Asian |
| Age — (median) years | 31 |
| Male sex — number (%) | 16(64%) |
| Bone marrow blasts at diagnosis— % | 83.56±10.91 |
| Subtype | B-lineage |
| White cell count — per mm^3^ |  |
| Max  Mean  Min | 237 800  65 703 ± 64 010  2 060 |
| Normal karyotype— % | 80% |
| Normal cytogenetic profile— % | 36% |

Plus-minus values are means± s.d.
