# Supplementary material for: Heterogeneity of CD34 and CD38 expression in acute B lymphoblastic leukemia cells is reversible and not hierarchically organized
Source: J Hematol Oncol. 2016 Sep 22;9:94. doi: 10.1186/s13045-016-0310-1 (PMC5034590; doi:10.1186/s13045-016-0310-1)
Supplement: Additional file 2: Table S2. — Clinical information of the 25 B-ALL patients. (DOCX 25 kb) [file 13045_2016_310_MOESM2_ESM.docx]

**Table S2. Clinical information of the 25 B-ALL patients.**

| Patient | Sex | Age | WBC | LDH  (U/L) | Spleno  megaly | Risk | Status of d28 | Xenografts |
| --- | --- | --- | --- | --- | --- | --- | --- | --- |
| ID |  | (year) | (10^9^/L) |  |  |  |  |  |
| #1 | Male | 20 | 46.3 | 247.5 | - | High | NR | Success |
| #2 | Female | 17 | 12.1 | 9022 | + | High | NR | Success |
| #3 | Male | 58 | 135.51 | 1965.5 | + | High | NR | Success |
| #4 | Male | 21 | 56 |  |  | High | NR | Success |
| #5 | Male | 20 | 228.07 | 580.1 | - | High | NR | No |
| #6 | Male | 47 | 100 | 434.4 | - | High | NR | No |
| #7 | Male | 20 | 2.06 |  |  | Standard | CR | Success |
| #8 | Female | 31 | 15.31 | 359.1 | + | Standard | NR | No |
| #9 | Male | 40 | 47.4 | 533 | - | High | NR | No |
| #10 | Male | 34 | 70.6 | 10000 | + | High | NR | Success |
| #11 | Female | 38 | 80 |  |  | High | NR | No |
| #12 | Female | 57 | 30 | 236 | - | High | NR | No |
| #13 | Female | 19 | 10.89 | 1791 | - | High | NR | Success |
| #14 | Male | 42 | 105.91 | 1135 | + | High | NR | Success |
|  |  |  |  |  |  |  |  |  |
| #15 | Female | 25 | 30 | 207.8 | - | High | NR | No |
| #16 | Female | 43 | 80 | 357.9 | - | High | NR | No |
| #17 | Male | 19 | 99.62 | 285.9 | + | High | NR | No |
| #18 | Male | 21 | 10.2 | 772.9 | - | Standard | CR | No |
| #19 | Male | 35 | 66 | 184.4 | + | High | NR | No |
| #20 | Male | 20 | 2.8 | 157 | - | High | NR | Success |
| #21 | Male | 58 | 85.2 | 941 | - | High | NR | No |
|  |  |  |  |  |  |  |  |  |
| #22 | Male | 32 | 20.2 | 1040.6 | + | High | NR | No |
| #23 | Male | 21 | 4.9 | 213.7 | - | Standard | CR | Success |
| #24 | Female | 19 | 237.8 | 1164.6 | + | High | NR | Success |
|  |  |  |  |  |  |  |  |  |
| #25 | Female | 38 | 20.6 |  |  | High | NR | Success |

WBC: white blood cell,

Risk of adult B-ALL is based on age, WBC, immunophenotypes, and cytogenetic aberrations.

CR means complete-remission, NR means non-remission.

Xenograft success means that B-ALL can be reconstituted in NSI mice.
