# Supplementary material for: Heterogeneity of CD34 and CD38 expression in acute B lymphoblastic leukemia cells is reversible and not hierarchically organized
Source: J Hematol Oncol. 2016 Sep 22;9:94. doi: 10.1186/s13045-016-0310-1 (PMC5034590; doi:10.1186/s13045-016-0310-1)
Supplement: Additional file 3: Table S3. — Summary of mice engrafted with different subpopulations of primary B-ALL cells from xenografts. (DOCX 31 kb) [file 13045_2016_310_MOESM3_ESM.docx]

**Table S3. Summary of mice engrafted with different subpopulations of primary B-ALL cells from xenografts.**

| Patient | Mouse ID | Generation | Subpopulation | Purity (%) | Dosage | Final Engraftment Level in BM(%) | Survival (weeks) |
| --- | --- | --- | --- | --- | --- | --- | --- |
| #1 | 131012N1 | 3rd | CD34+CD38- | 98.2 | 1.0 x 10^4^ | 50.1 | 14 |
|  | 131012N2 | 3rd | CD34+CD38- | 98.2 | 1.0 x 10^4^ | 0 | 2 |
|  | 131012N3 | 3rd | CD34+CD38- | 98.2 | 1.0 x 10^4^ | 0 | 16 |
|  | 131012N4 | 3rd | CD34+CD38- | 98.2 | 1.0 x 10^4^ | 94.8 | 14 |
|  | 131012N5 | 3rd | CD34+CD38- | 98.2 | 1.0 x 10^4^ | 98.5 | 14 |
|  | 131012N6 | 3rd | CD34+CD38+ | 96.1 | 1.0 x 10^4^ | 0 | 14 |
|  | 131012N7 | 3rd | CD34+CD38+ | 96.1 | 1.0 x 10^4^ | 94.8 | 18 |
|  | 131012N8 | 3rd | CD34+CD38+ | 96.1 | 1.0 x 10^4^ | 96.1 | 14 |
|  | 131012N9 | 3rd | CD34-CD38+ | 96.4 | 1.0 x 10^4^ | 0 | 2 |
|  | 131012N10 | 3rd | CD34-CD38+ | 96.4 | 1.0 x 10^4^ | 97.8 | 14 |
|  | 131012N11 | 3rd | CD34-CD38+ | 96.4 | 1.0 x 10^4^ | 97.8 | 14 |
|  | 131129N1 | 4th | CD34+CD38- | 97.8 | 4.0 x 10^3^ | 61.8 | 13 |
|  | 131129N2 | 4th | CD34+CD38- | 97.8 | 4.0 x 10^3^ | 0 | 2 |
|  | 131129N3 | 4th | CD34+CD38- | 97.8 | 4.0 x 10^3^ | 91.1 | 16 |
|  | 131129N4 | 4th | CD34+CD38- | 97.8 | 4.0 x 10^3^ | 36.4 | 15 |
|  | 131129N5 | 4th | CD34+CD38- | 97.8 | 4.0 x 10^3^ | 85.1 | 16 |
|  | 131129N6 | 4th | CD34-CD38+ | 96.6 | 4.0 x 10^3^ | 91.1 | 16 |
|  | 131129N7 | 4th | CD34-CD38+ | 96.6 | 4.0 x 10^3^ | 41.7 | 14 |
|  | 131129N8 | 4th | CD34-CD38+ | 96.6 | 4.0 x 10^3^ | 46.4 | 15 |
|  | 131129N9 | 4th | CD34-CD38+ | 96.6 | 4.0 x 10^3^ | 93.4 | 17 |
|  | 131129N10 | 4th | CD34-CD38+ | 96.6 | 4.0 x 10^3^ | 92.1 | 16 |
|  | 131129N11 | 4th | CD34+CD38+ | 97.2 | 4.0 x 10^3^ | 90.4 | 16 |
|  | 131129N12 | 4th | CD34+CD38+ | 97.2 | 4.0 x 10^3^ | 60.9 | 17 |
|  | 131129N13 | 4th | CD34+CD38+ | 97.2 | 4.0 x 10^3^ | 0 | 1 |
|  | 131129N14 | 4th | CD34+CD38+ | 97.2 | 4.0 x 10^3^ | 94.7 | 16 |
|  | 131129N15 | 4th | CD34+CD38+ | 97.2 | 4.0 x 10^3^ | 82.3 | 19 |
|  |  |  |  |  |  |  |  |
| #3 | 131224A1 | 3rd | CD34-CD38+ | 98 | 1 X 10^2^ | 51.8 | 14 |
|  | 131224A2 | 3rd | CD34-CD38+ | 98 | 1 X 10^2^ | 96.7 | 21 |
|  | 131224A3 | 3rd | CD34-CD38+ | 98 | 1 X 10^2^ | 0 | 14 |
|  | 131224A4 | 3rd | CD34-CD38+ | 98 | 1 x 10^3^ | 0 | 14 |
|  | 131224A5 | 3rd | CD34-CD38+ | 98 | 1 x 10^3^ | 89.7 | 14 |
|  | 131224A6 | 3rd | CD34-CD38+ | 98 | 1 x 10^3^ | 82.4 | 14 |
|  | 131224A7 | 3rd | CD34-CD38+ | 98 | 1 x 10^4^ | 85.4 | 12 |
|  | 131224A8 | 3rd | CD34-CD38+ | 98 | 1 x 10^4^ | 90.2 | 13 |
|  | 131224A9 | 3rd | CD34-CD38+ | 98 | 1 x 10^4^ | 53.4 | 12 |
|  | 131224A10 | 3rd | CD34-CD38+ | 98 | 1 x 10^4^ | 87.4 | 10 |
|  | 131224B1 | 3rd | CD34+CD38+ | 96.4 | 1 X 10^2^ | 15.2 | 14 |
|  | 131224B2 | 3rd | CD34+CD38+ | 96.4 | 1 X 10^2^ | 47.8 | 16 |
|  | 131224B3 | 3rd | CD34+CD38+ | 96.4 | 1 X 10^2^ | 0 | 16 |
|  | 131224B4 | 3rd | CD34+CD38+ | 96.4 | 1 x 10^3^ | 0 | 16 |
|  | 131224B5 | 3rd | CD34+CD38+ | 96.4 | 1 x 10^3^ | 27.1 | 14 |
|  | 131224B6 | 3rd | CD34+CD38+ | 96.4 | 1 x 10^3^ | 80 | 14 |
|  | 131224B7 | 3rd | CD34+CD38+ | 96.4 | 1 x 10^4^ | 0 | 2 |
|  | 131224B8 | 3rd | CD34+CD38+ | 96.4 | 1 x 10^4^ | 82.6 | 14 |
|  | 131224B9 | 3rd | CD34+CD38+ | 96.4 | 1 x 10^4^ | 0 | 16 |
|  | 131224B10 | 3rd | CD34+CD38+ | 96.4 | 1 x 10^4^ | 53.4 | 16 |
|  |  |  |  |  |  |  |  |
| #4 | 131009N1 | 3rd | CD34+CD38+ | 98.6 | 1 X 10^2^ | 84.5 | 14 |
|  | 131009N2 | 3rd | CD34+CD38+ | 98.6 | 1 X 10^2^ | 90.1 | 14 |
|  | 131009N3 | 3rd | CD34+CD38+ | 98.6 | 1 x 10^3^ | 93.6 | 11 |
|  | 131009N4 | 3rd | CD34+CD38+ | 98.6 | 1 x 10^3^ | 89.6 | 12 |
|  | 131009N5 | 3rd | CD34+CD38+ | 98.6 | 1 x 10^4^ | 85 | 14 |
|  | 131009N6 | 3rd | CD34+CD38+ | 98.6 | 1 x 10^4^ | 91.6 | 10 |
|  | 131009N7 | 3rd | CD34-CD38+ | 97.8 | 1 x 10^3^ | 0 | 16 |
|  | 131009N8 | 3rd | CD34-CD38+ | 97.8 | 1 x 10^3^ | 0 | 2 |
|  | 131009N9 | 3rd | CD34-CD38+ | 97.8 | 1 x 10^3^ | 56.4 | 14 |
|  | 131009N10 | 3rd | CD34-CD38+ | 97.8 | 1 x 10^4^ | 0 | 14 |
|  | 131009N11 | 3rd | CD34-CD38+ | 97.8 | 1 x 10^4^ | 86.4 | 16 |
|  | 131009N12 | 3rd | CD34-CD38+ | 97.8 | 1 x 10^4^ | 56.4 | 14 |

The table shows the sorted B-ALL subpopulations from the 3 engrafting leukemia (patient #1, 3, 4). B-ALL cells from patient #1 contain CD34-CD38+, CD34+CD38+, and CD34-CD38+ subpopulations; B-ALL cells from patients #3 and #4 contain CD34-CD38+ and CD34+CD38+ subpopulations.
