# Supplementary figures and images for: Heterogeneity of CD34 and CD38 expression in acute B lymphoblastic leukemia cells is reversible and not hierarchically organized
Source: J Hematol Oncol. 2016 Sep 22;9:94. doi: 10.1186/s13045-016-0310-1 (PMC5034590; doi:10.1186/s13045-016-0310-1)

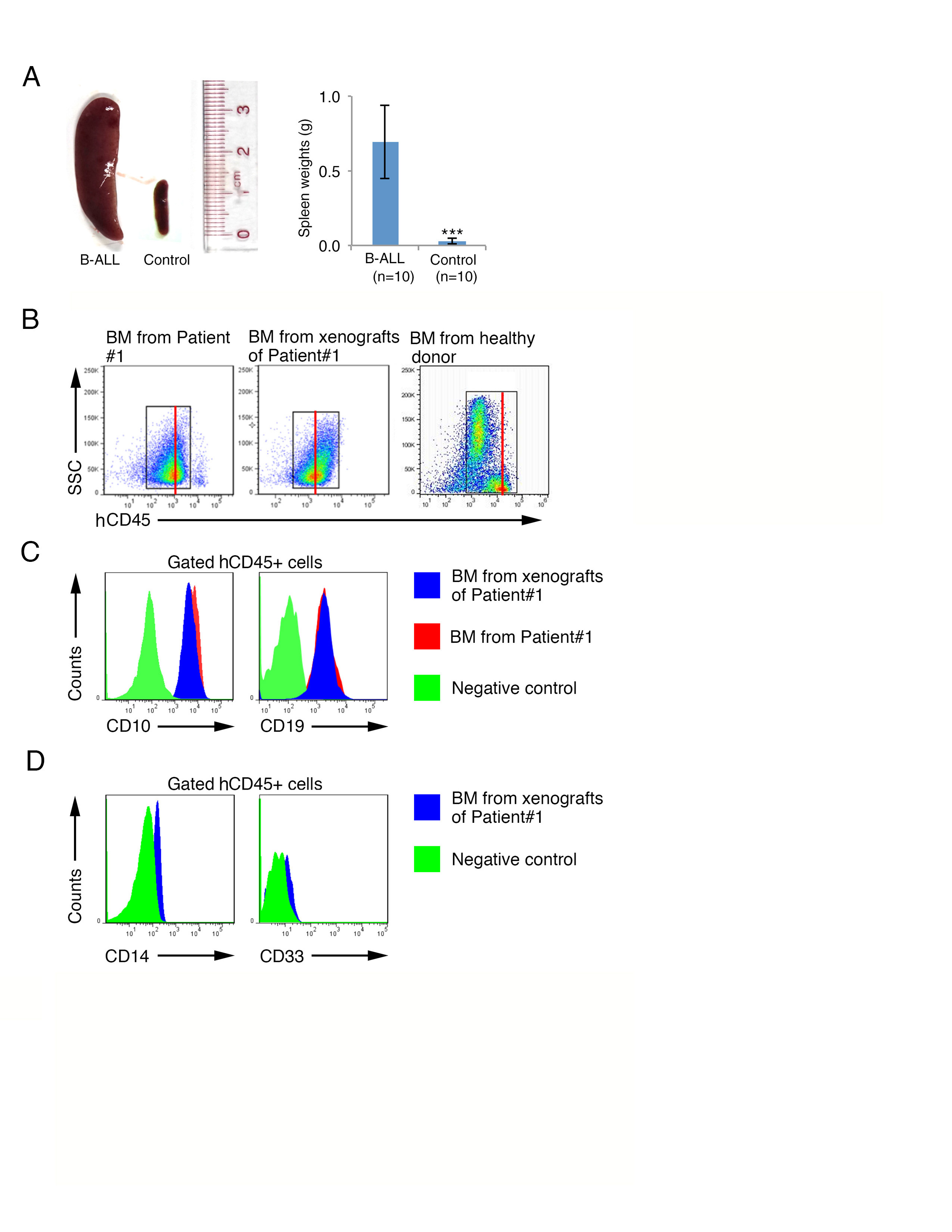

Supplement: Additional file 4: Figure S1. — Reconstitution of adult B-ALL in NSI mice. (A) Left, spleens from of an NSI mouse engrafted with B-ALL cells and an NSI mouse not injected with B-ALL cells. Right, weights of the spleens from the mice engrafted with or without B-ALL (0.69 ± 0.24 versus 0.02 ± 0.02; p = 0.001). Data are shown as mean ± SEM. (B) Representative FACS analysis of primary leukemic cells from patient #1, BM cells from xenografts of patient #1 (mouse #140815N1), and BM cells from a healthy donor. Red lines highlight the median values of hCD45 expression levels in leukemic cells and in lymphoid cells. (C) Representative FACS analysis of hCD10 and CD19 cells from patient #1 and leukemic cells from xenograft of patient #1 (mouse #140815N1). (D) Representative FACS analysis of hCD14 and hCD33 cells from BM cells from xenograft of patient #1 (mouse #140815N1). (JPG 648 kb) [file 13045_2016_310_MOESM4_ESM.jpg]

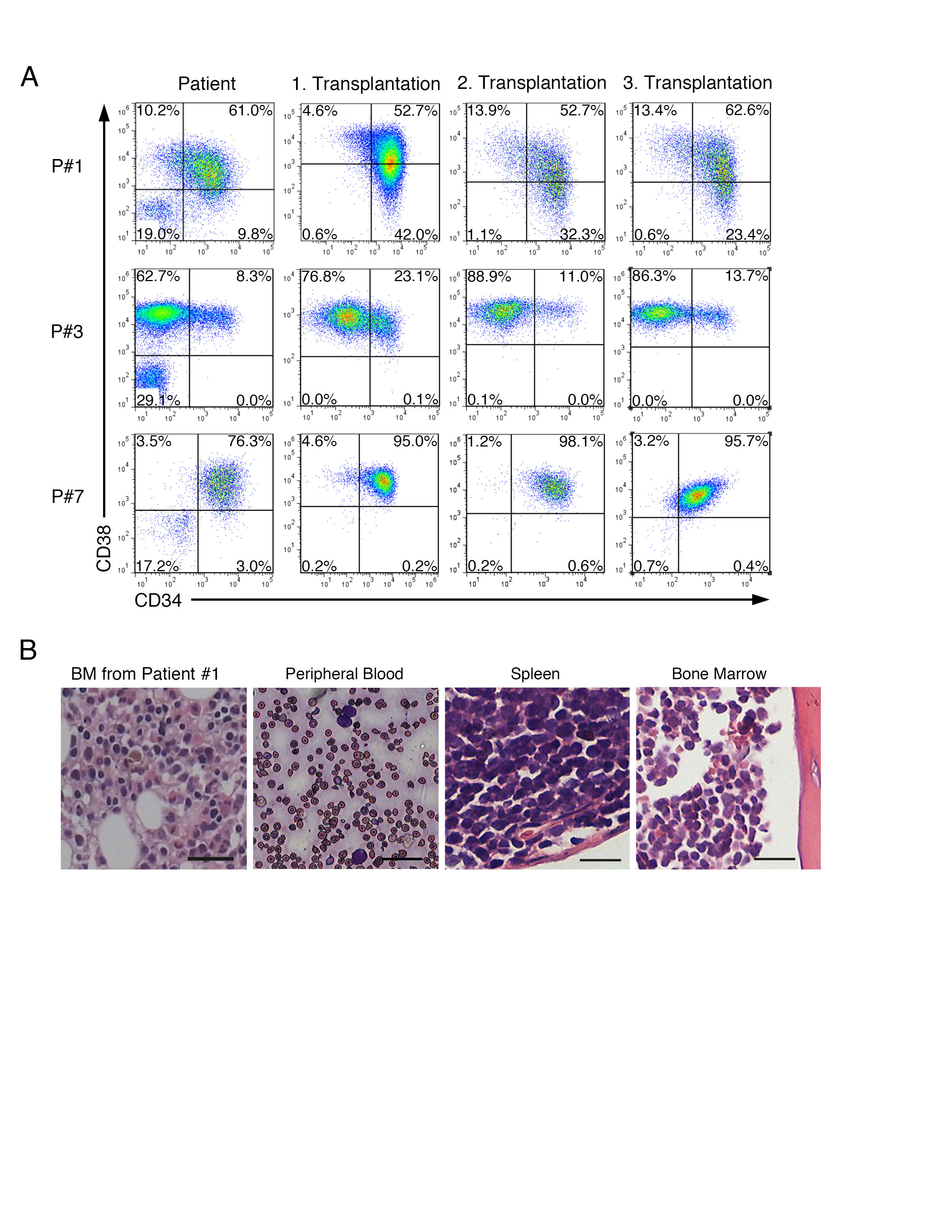

Supplement: Additional file 5: Figure S2. — Immunophenotypes of the leukemic cells remain stable for serial transplantations. (A) Representative FACS analysis of leukemic cells from patients #1, #3, and #7 and leukemic cells from their xenografts after serial transplantations. (B) Representative of H&E staining of blood smear (peripheral blood), spleen and bone marrow of xenografts of patient #1 and patient #1 BM. Scale bars represent 100 μm (JPG 1500 kb) [file 13045_2016_310_MOESM5_ESM.jpg]

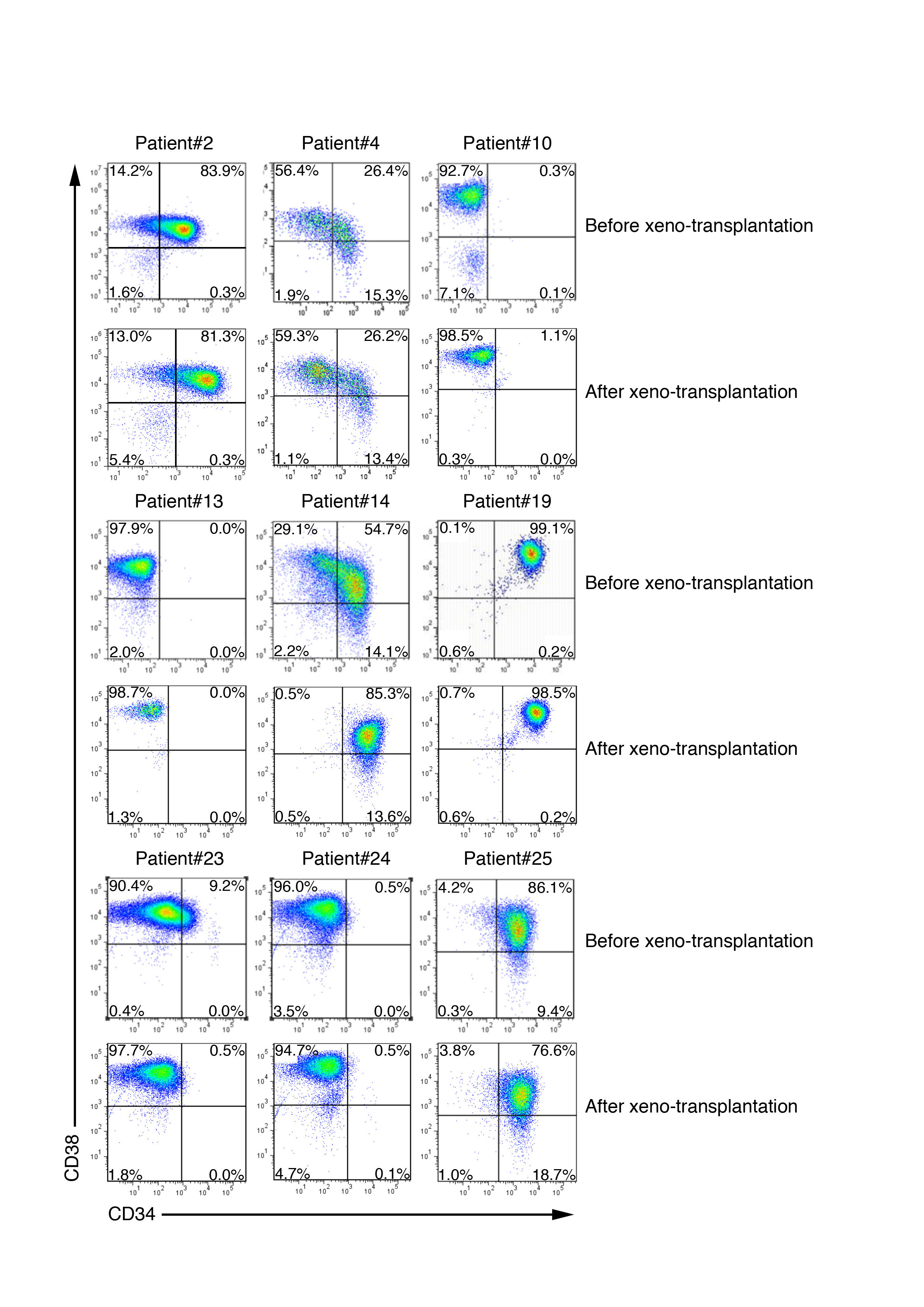

Supplement: Additional file 6: Figure S3. — Characterizations of CD34 and CD38 expression profiles in B-ALL cells in patients and xenografts. BM cells of xenografts were analyzed by FACS to determine their expression profiles of CD34 and CD38. (JPG 1305 kb) [file 13045_2016_310_MOESM6_ESM.jpg]

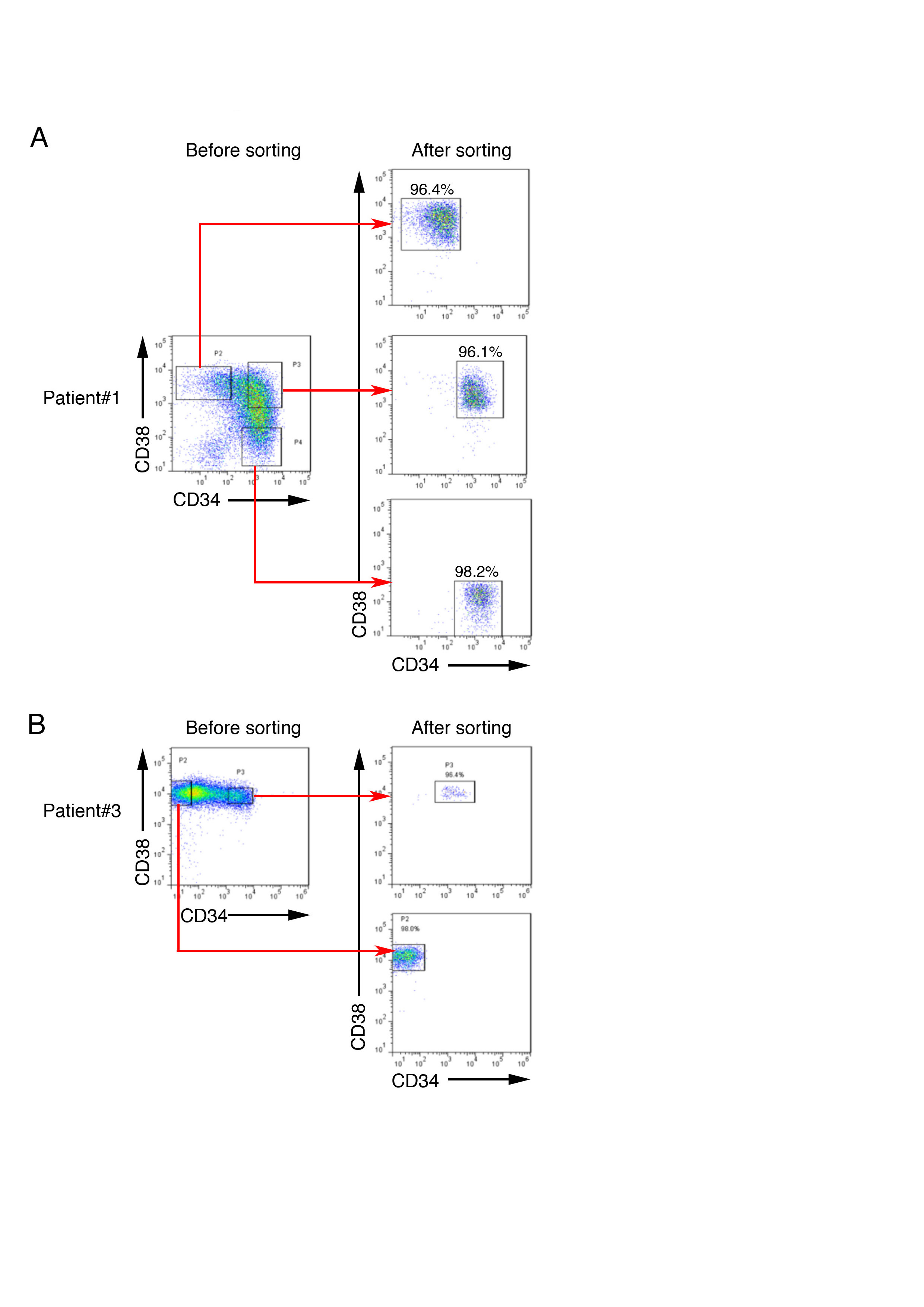

Supplement: Additional file 7: Figure S4. — Reevaluation of purities of primary B-ALL cells after sorting. (A) Representative FACS analysis of sorted CD34+CD38−, CD34+CD38+, and CD34−CD38+ fractions from xenograft of patient #1. (B) Representative FACS analysis of purified CD34+CD38+ and CD34−CD38+ fractions from xenograft of patient #3. (JPG 550 kb) [file 13045_2016_310_MOESM7_ESM.jpg]

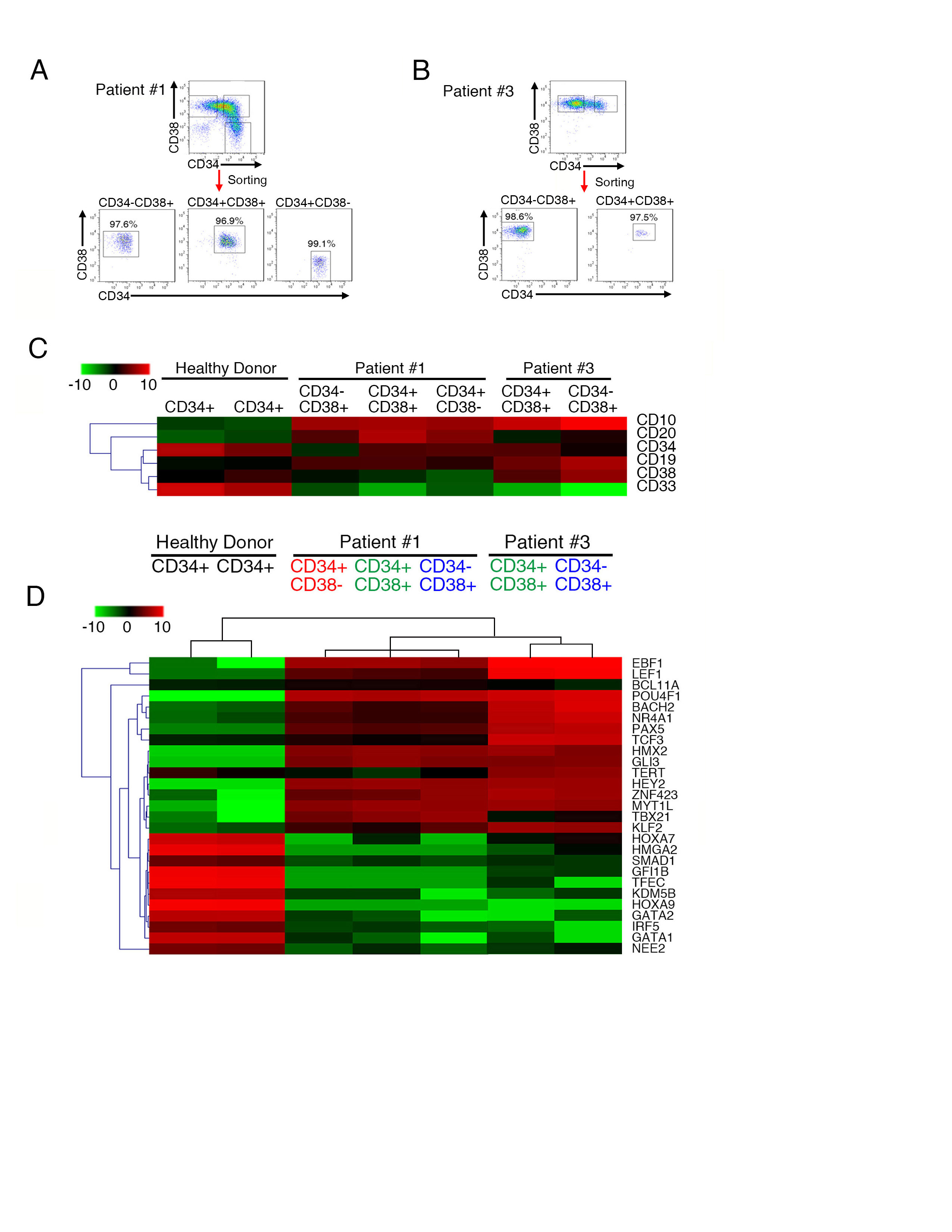

Supplement: Additional file 8: Figure S5. — Gene expression pattern in subpopulations of B-ALL cells. (A, B) The immunophenotypes of patients #1 and #3 respected to CD34 and CD38 expression for RNA-Seq analysis. (C) The expression levels of CD10, CD20, CD34, CD19, CD38, and CD33 in indicated subpopulations from patients #1, #3, and two healthy donors. (D) Hierarchical clustering shows different subpopulations of patient #1 and #3 are grouped together. (JPG 758 kb) [file 13045_2016_310_MOESM8_ESM.jpg]

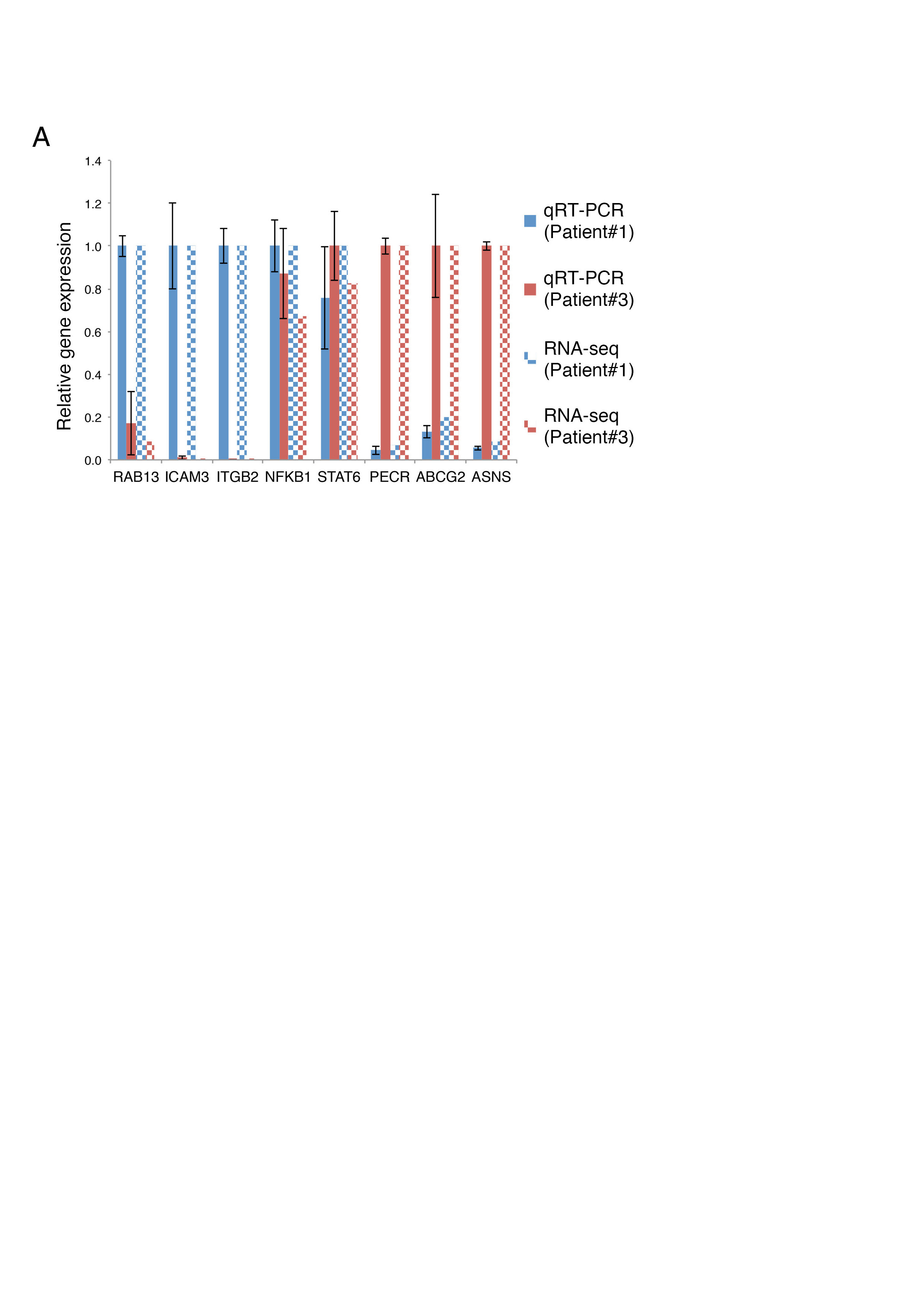

Supplement: Additional file 9: Figure S6. — The expression level of genes was confirmed by qRT-PCR. (A) Relative expression levels of indicated genes in patient #1 (blue) and patient #3 (red) cells were measured by qRT-PCR. The results were normalized to β-ACTIN mRNA levels and represent the means ± SEM. (n = 3). The qRT-PCR results were compared to the expression levels of these genes in B-ALL cells from patient #1 and patient #3 indicated by RNA-Seq analysis. (JPG 516 kb) [file 13045_2016_310_MOESM9_ESM.jpg]

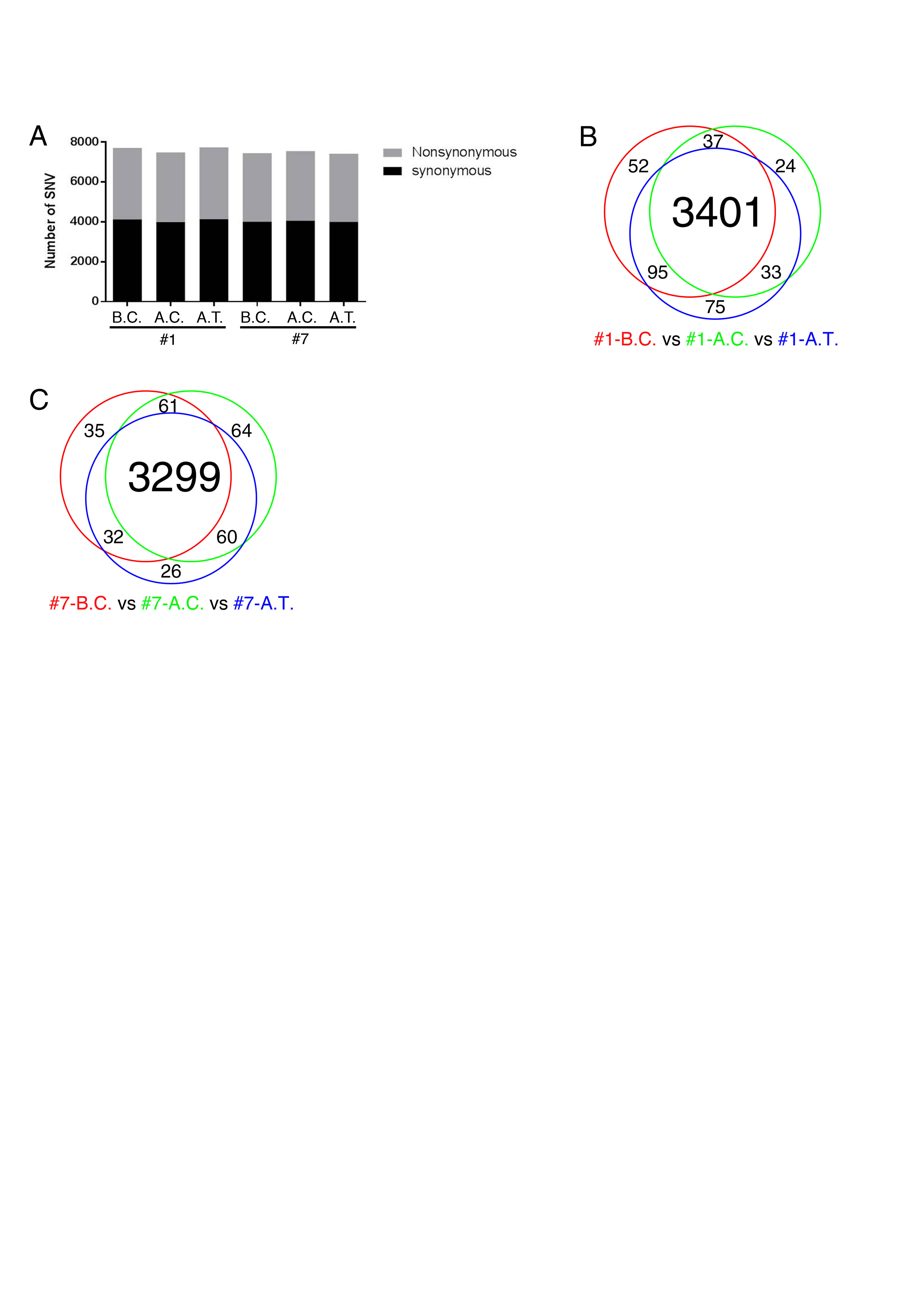

Supplement: Additional file 12: Figure S7. — Leukemic cells’ SNPs are conserved in ex vivo. (A) Whole exome sequencing of the leukemia in xenografts (before culture, B. C.), after culture (A. C.) and after transplantation (A. T.) revealed the presence of genome mutations. Similar rates of nonsynonymous and synonymous single nucleotide variants (SNVs) within the leukemia of patient #1 and patient #7. (B, C) Venn diagram present B-ALL cells from co-culture and B-ALL cells from xenografts share similar nonsynonymous SNP profiles. (JPG 1513 kb) [file 13045_2016_310_MOESM12_ESM.jpg]

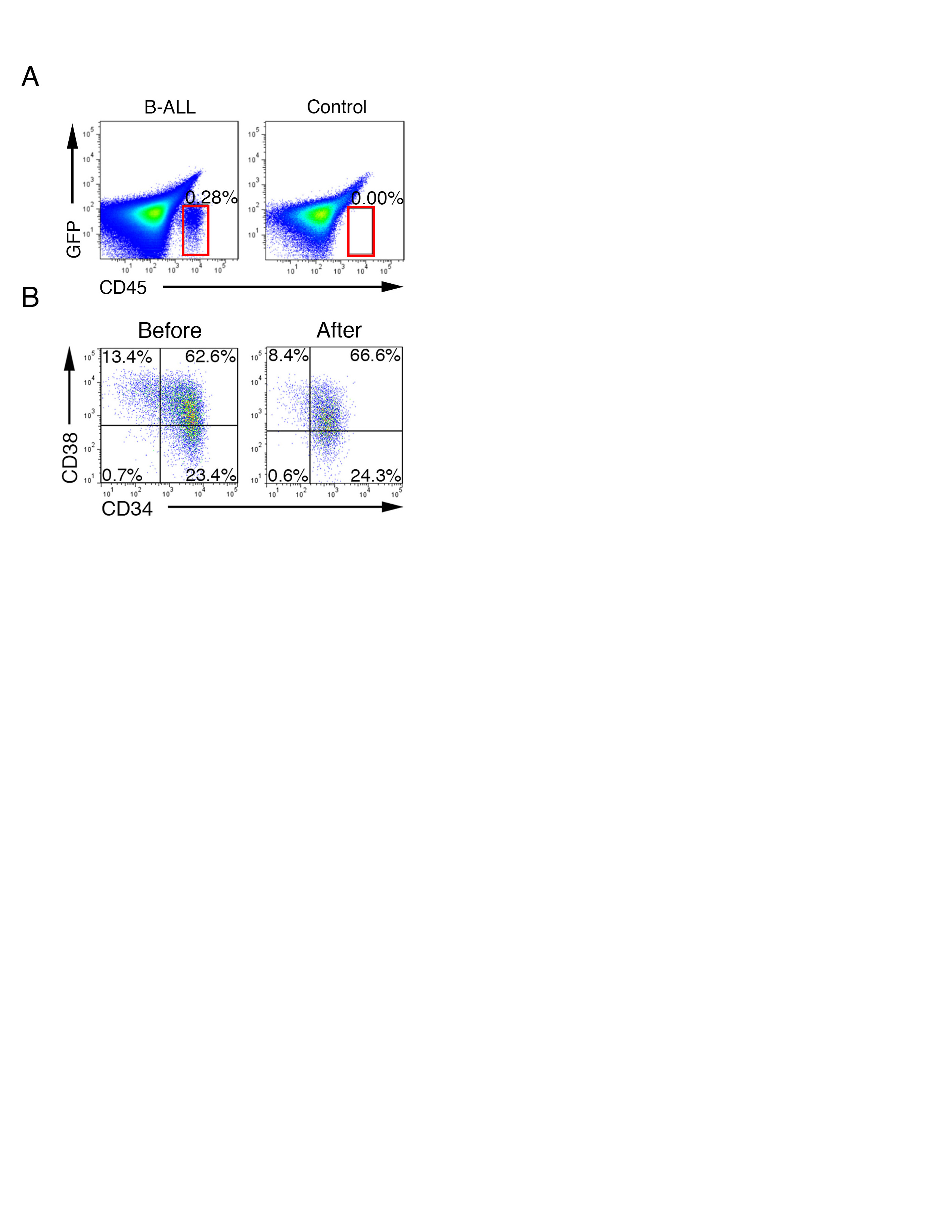

Supplement: Additional file 13: Figure S8. — Single cell assays of primary adult B-ALL cells. (A) Each NSI mouse was injected with a single primary B-ALL cell. Sixteen weeks after transplantation, the BM compartments of hosts were subjected to FACS analysis. (B) Representative FACS analysis of B-ALL cells used for single cell transplantation (Before, left) and gated hCD45+ cells from the BM compartments of the successfully grafted hosts (After, right). (JPG 494 kb) [file 13045_2016_310_MOESM13_ESM.jpg]
