# Supplementary material for: Heterogeneity of CD34 and CD38 expression in acute B lymphoblastic leukemia cells is reversible and not hierarchically organized
Source: J Hematol Oncol. 2016 Sep 22;9:94. doi: 10.1186/s13045-016-0310-1 (PMC5034590; doi:10.1186/s13045-016-0310-1)
Supplement: Additional file 10: Table S4. — Characteristics of leukemic cells in co-culture with OP9 stromal cells. (DOCX 18 kb) [file 13045_2016_310_MOESM10_ESM.docx]

**Table S4. Characteristics of leukemic cells in co-culture with OP9 stromal cells.**

| Patient | Adhesion | Growth | Final  Immunophenotypes | Culture Time (month) |
| --- | --- | --- | --- | --- |
| #1 | Yes | Yes | CD34-CD38+ | 6 |
| #2 | Yes | Yes | CD34-CD38+ | 2 |
| #4 | Yes | Yes | CD34-CD38+ | 3 |
| #7 | Yes | Yes | CD34-CD38+ | 2 |
| #10 | Yes | Yes | CD34-CD38+ | 3 |
| #13 | Yes | Yes | CD34-CD38+ | 2 |
| #14 | Yes | Yes | CD34-CD38+ | 2 |
| #19 | Yes | Yes | CD34-CD38+ | 2 |
| #23 | Yes | Yes | CD34-CD38+ | 3 |
| #24 | Yes | Yes | CD34-CD38+ | 2 |
| #25 | Yes | Yes | CD34-CD38+ | 4 |

11 of 12 engrafting leukemic cells attached to OP9 cells and grew robustly in the co-culture from 2 to 6 months. FACS analysis of CD34 and CD38 expression profiles show that leukemic cells from the 11 patients gradually downregulated CD34 expression.
