# Supplementary material for: Heterogeneity of CD34 and CD38 expression in acute B lymphoblastic leukemia cells is reversible and not hierarchically organized
Source: J Hematol Oncol. 2016 Sep 22;9:94. doi: 10.1186/s13045-016-0310-1 (PMC5034590; doi:10.1186/s13045-016-0310-1)
Supplement: Additional file 11: Table S5. — Engrafted mice transplanted with cultured leukemic cells. (DOCX 19 kb) [file 13045_2016_310_MOESM11_ESM.docx]

**Table S5. Engrafted mice transplanted with cultured leukemic cells.**

| Patient | Mouse ID | Culture Time (months) | Subpopulation | Dosage | Final Engraftment Level (%) | Survival Time (weeks) |
| --- | --- | --- | --- | --- | --- | --- |
| #1 | 131123F2 | 4 | CD34-CD38+ | 1 x 10^5^ | 88.4 | 12 |
| #1 | 131123F6 | 4 | CD34-CD38+ | 1 x 10^5^ | 94.4 | 12 |
| #1 | 131123F8 | 4 | CD34-CD38+ | 1 x 10^5^ | 91.2 | 11 |
| #1 | 131123F11 | 4 | CD34-CD38+ | 1 x 10^5^ | 89.6 | 11 |
| #1 | 131123F12 | 4 | CD34-CD38+ | 1 x 10^5^ | 80.2 | 11 |
| #4 | 131107F1 | 2 | CD34-CD38+ | 2 x 10^5^ | 69.1 | 11 |
| #4 | 131107F2 | 2 | CD34-CD38+ | 2 x 10^5^ | 66.2 | 11 |
| #4 | 131107F3 | 2 | CD34-CD38+ | 2 x 10^5^ | 73.7 | 12 |
| #4 | 131107F4 | 2 | CD34-CD38+ | 2 x 10^5^ | 63.9 | 10 |
| #4 | 131107F5 | 2 | CD34-CD38+ | 2 x 10^5^ | 0 | 4 |
| #7 | 140228nN1 | 2 | CD34-CD38+ | 1 x 10^5^ | 30 | 8 |
| #7 | 140228nN2 | 2 | CD34-CD38+ | 1 x 10^5^ | 91.2 | 12 |
| #7 | 140228nN3 | 2 | CD34-CD38+ | 1 x 10^5^ | 94.6 | 12 |
| #7 | 140228nN4 | 2 | CD34-CD38+ | 1 x 10^5^ | 0 | 2 |

The table shows the mice that were transplanted with in vitro expanded leukemic cells that were CD34-CD38+ co-culture with OP9 stromal cells from patients #1, #4, and #7.
