# Supplementary material for: Heterogeneity of CD34 and CD38 expression in acute B lymphoblastic leukemia cells is reversible and not hierarchically organized
Source: J Hematol Oncol. 2016 Sep 22;9:94. doi: 10.1186/s13045-016-0310-1 (PMC5034590; doi:10.1186/s13045-016-0310-1)
Supplement: Additional file 14: Table S6. — Summary of single B-ALL cell xenotransplantation. (DOCX 27 kb) [file 13045_2016_310_MOESM14_ESM.docx]

**Table S6. Summary of single B-ALL cell xenotransplantation.**

| Patient | Mice ID | Generation | Dosage | Final  Engraftment Level (%) | Survival (weeks) |
| --- | --- | --- | --- | --- | --- |
| #1 | 140127A1 | 1st | 1 | 0 | 14 |
| #1 | 140127A2 | 1st | 1 | 0 | 14 |
| #1 | 140127A3 | 1st | 1 | 0 | 14 |
| #1 | 140127A4 | 1st | 1 | 0 | 14 |
| #1 | 140127A5 | 1st | 1 | 0 | 14 |
| #1 | 140127A6 | 1st | 1 | 0 | 14 |
| #1 | 140127A7 | 1st | 1 | 0 | 14 |
| #1 | 140127A8 | 1st | 1 | 0 | 14 |
| #1 | 140127A9 | 1st | 1 | 0 | 14 |
| #1 | 140127A10 | 1st | 1 | 0 | 14 |
| #1 | 140127A11 | 1st | 1 | 0 | 14 |
| #1 | 140127A12 | 1st | 1 | 0 | 14 |
| #1 | 140127B1 | 1st | 1 | 0 | 14 |
| #1 | 140127B2 | 1st | 1 | 0 | 14 |
| #1 | 140127B3 | 1st | 1 | 0 | 14 |
| #1 | 140127B4 | 1st | 1 | 0 | 14 |
| #1 | 140127B5 | 1st | 1 | 0 | 14 |
| #1 | 140127B6 | 1st | 1 | 0 | 14 |
| #1 | 140127B7 | 1st | 1 | 0 | 14 |
| #1 | 140127B8 | 1st | 1 | 0 | 14 |
| #1 | 140127B9 | 1st | 1 | 0 | 14 |
| #1 | 140127B10 | 1st | 1 | 0 | 14 |
| #1 | 140127C1 | 1st | 1 | 0 | 15 |
| #1 | 140127C2 | 1st | 1 | 0 | 15 |
| #1 | 140127C3 | 1st | 1 | 0 | 15 |
| #1 | 140127C4 | 1st | 1 | 0 | 15 |
| #1 | 140127C5 | 1st | 1 | 0 | 15 |
| #1 | 140127C6 | 1st | 1 | 0.12 | 15 |
| #1 | 140127C7 | 1st | 1 | 0 | 15 |
| #1 | 140127C8 | 1st | 1 | 0 | 15 |
| #1 | 140127C9 | 1st | 1 | 0 | 15 |
| #1 | 140127C10 | 1st | 1 | 0 | 15 |
| #1 | 140127D1 | 1st | 1 | 0 | 15 |
| #1 | 140127D2 | 1st | 1 | 0 | 15 |
| #1 | 140127D3 | 1st | 1 | 0 | 15 |
| #1 | 140127D4 | 1st | 1 | 0 | 15 |
| #1 | 140127D5 | 1st | 1 | 0.26 | 15 |
| #1 | 140127D6 | 1st | 1 | 0 | 15 |
| #1 | 140127D7 | 1st | 1 | 0 | 15 |
| #1 | 140127D8 | 1st | 1 | 0 | 15 |
| #1 | 140127D9 | 1st | 1 | 0 | 15 |
| #1 | 140127D10 | 1st | 1 | 0 | 15 |
| #1 | 140127E1 | 1st | 1 | 0 | 15 |
| #1 | 140127E2 | 1st | 1 | 0 | 15 |
| #1 | 140127E3 | 1st | 1 | 0 | 15 |
| #1 | 140127E4 | 1st | 1 | 0 | 15 |
| #1 | 140127E5 | 1st | 1 | 0 | 15 |
| #1 | 140127E6 | 1st | 1 | 0 | 15 |
| #1 | 140127E7 | 1st | 1 | 0 | 15 |
| #1 | 140127E8 | 1st | 1 | 0 | 15 |
| #1 | 140127E9 | 1st | 1 | 0 | 15 |
| #1 | 140127E10 | 1st | 1 | 0 | 15 |
| #1 | 140127F1 | 1st | 1 | 0 | 16 |
| #1 | 140127F2 | 1st | 1 | 0 | 16 |
| #1 | 140127F3 | 1st | 1 | 0 | 16 |
| #1 | 140127F4 | 1st | 1 | 0.45 | 16 |
| #1 | 140127F5 | 1st | 1 | 0 | 16 |
| #1 | 140127F6 | 1st | 1 | 0 | 16 |
| #1 | 140127F7 | 1st | 1 | 0 | 16 |
| #1 | 140127F8 | 1st | 1 | 0 | 16 |
| #1 | 140127F9 | 1st | 1 | 0 | 16 |
| #1 | 140127F10 | 1st | 1 | 0 | 16 |
| #1 | 140127G1 | 1st | 1 | 0 | 16 |
| #1 | 140127G2 | 1st | 1 | 0 | 16 |
| #1 | 140127G3 | 1st | 1 | 0 | 16 |
| #1 | 140127G4 | 1st | 1 | 0 | 16 |
| #1 | 140127G5 | 1st | 1 | 0 | 16 |
| #1 | 140127G6 | 1st | 1 | 0.28 | 16 |
| #1 | 140127G7 | 1st | 1 | 0 | 16 |
| #1 | 140127G8 | 1st | 1 | 0 | 16 |

Single B-ALL cells from patient #1 isolated by serial dilution were confirmed by microscopy and were injected into NSI mice. The recipient mice were killed for detection of B-ALL reconstitution 14-16 weeks after transplantation.
