# Supplementary material for: Heterogeneity of CD34 and CD38 expression in acute B lymphoblastic leukemia cells is reversible and not hierarchically organized
Source: J Hematol Oncol. 2016 Sep 22;9:94. doi: 10.1186/s13045-016-0310-1 (PMC5034590; doi:10.1186/s13045-016-0310-1)
Supplement: Additional file 15: Table S7. — Antibodies used for multicolor FACS analysis and cell sorting. (DOCX 19 kb) [file 13045_2016_310_MOESM15_ESM.docx]

**Table S7. Antibodies used for multicolor FACS analysis and cell sorting.**

| Name | Clone | Supplier |
| --- | --- | --- |
| CD34 APC | 4H11 | eBioscience, San Diego, CA, USA |
| CD38 PE | HIT2 | eBioscience, San Diego, CA, USA |
| CD45 PerCP-Cyanine5.5 | HI30 | eBioscience, San Diego, CA, USA |
| CD19 APC-eFluor® 780 | HIB19 | eBioscience, San Diego, CA, USA |
| CD10 FITC | SN5c | eBioscience, San Diego, CA, USA |
| CD20 eFluor 450 | 2H7 | eBioscience, San Diego, CA, USA |
| CD14 PE | 61D3 | eBioscience, San Diego, CA, USA |
| CD33 APC | P67.6 | eBioscience, San Diego, CA, USA |
| CD3 FITC | OKT3 | eBioscience, San Diego, CA, USA |
| muCD19 APC | MB19-1 | eBioscience, San Diego, CA, USA |
| muB220 PE | RA3-6B2 | eBioscience, San Diego, CA, USA |
| muCD3 FITC | 145-2C11 | eBioscience, San Diego, CA, USA |
| muNKp46 PE | 29A1.4 | eBioscience, San Diego, CA, USA |
| muCD4PE | RM4-5 | eBioscience, San Diego, CA, USA |
| mCD8 PerCP-Cyanine5.5 | 53-6.7 | eBioscience, San Diego, CA, USA |
