# Supplementary material for: Heterogeneity of CD34 and CD38 expression in acute B lymphoblastic leukemia cells is reversible and not hierarchically organized
Source: J Hematol Oncol. 2016 Sep 22;9:94. doi: 10.1186/s13045-016-0310-1 (PMC5034590; doi:10.1186/s13045-016-0310-1)
Supplement: Additional file 16: Table S8. — Primers used for qRT-PCR and PCR. (DOCX 20 kb) [file 13045_2016_310_MOESM16_ESM.docx]

## Table S8. Primers used for qRT-PCR and PCR.

| Gene | Primer sequence | Fragment Size  (bp) | RefSeq ID |
| --- | --- | --- | --- |
| RAB13 | 5-AGCCGTGTCCCAGACTTGTA-3  5-TGTCTGATCATTCGCTTTGC-3 | 129 | [NM_002870](http://www.ncbi.nlm.nih.gov/entrez/query.fcgi?cmd=Search&db=Nucleotide&term=NM_002870) |
| ICAM3 | 5- CGCAGGGTGAAGTTCTGG-3  5-AGTGTACTGCAATGGCTCCC-3 | 123 | [NM_002162](http://www.ncbi.nlm.nih.gov/entrez/query.fcgi?cmd=Search&db=Nucleotide&term=NM_002162) |
| ITGB2 | 5-TGCTGACCTTGAACTTCGTG-3  5-GGACTCCAGCACACCGAG-3 | 116 | [NM_000211](http://www.ncbi.nlm.nih.gov/entrez/query.fcgi?cmd=Search&db=Nucleotide&term=NM_000211) |
| NFKB1 | 5-ATAACCTTTGCTGGTCCCAC-3  5-ATGTATGTGAAGGCCCATCC-3 | 112 | [NM_003998](http://www.ncbi.nlm.nih.gov/entrez/query.fcgi?cmd=Search&db=Nucleotide&term=NM_003998) |
| STAT6 | 5-CAGACCCCACAGAGACATGA-3  5-TCGCTGGACAGAGCTACAGA-3 | 118 | [NM_003153](http://www.ncbi.nlm.nih.gov/entrez/query.fcgi?cmd=Search&db=Nucleotide&term=NM_003153) |
| PECR | 5-TGCTTTGTGGGAGGTAGGTT-3  5-GAAAAGCCATCGTGAAGGAG-3 | 121 | [NM_018441](http://www.ncbi.nlm.nih.gov/entrez/query.fcgi?cmd=Search&db=Nucleotide&term=NM_018441) |
| ABCG2 | 5-AAGCCATTGGTGTTTCCTTG-3  5-CTGAGATCCTGAGCCTTTGG-3 | 123 | [NM_004827](http://www.ncbi.nlm.nih.gov/entrez/query.fcgi?cmd=Search&db=Nucleotide&term=NM_004827) |
| ASNS | 5-TGTCCAGGAAGAAAAGGCTC-3  5-CAGAAGATGGATTTTTGGCTG-3 | 103 | [NM_133436](http://www.ncbi.nlm.nih.gov/entrez/query.fcgi?cmd=Search&db=Nucleotide&term=NM_133436) |
| EBNA-2a | 5-AACTTCAACCCACACCATCA-3  5-TTCTGGACTATCTGGATCAT-3 | 116 | NC_007605.1 |
|  |  |  |  |
