# Supplementary material for: Heterogeneity of CD34 and CD38 expression in acute B lymphoblastic leukemia cells is reversible and not hierarchically organized
Source: J Hematol Oncol. 2016 Sep 22;9:94. doi: 10.1186/s13045-016-0310-1 (PMC5034590; doi:10.1186/s13045-016-0310-1)
Supplement: Additional file 17: — Supplementary methods (Additional file 15: Table S7 and Additional file 16: Table S8). (DOCX 44 kb) [file 13045_2016_310_MOESM17_ESM.docx]

**Supplementary Methods**

**Mice**

Animal experiments were performed in the Laboratory Animal Center of the Guangzhou Institutes of Biomedicine and Health (GIBH). We generated NOD-*scid*-*IL2Rg-/-*mice by TALEN-mediated gene targeting in the NOD background[1, 2]. Briefly, Female NOD/SCID mice (6-8 weeks of age, Charles River) were superovulated by intraperitoneal injection with 5 U of PMSG (sigma, cat.no. G4877-2000IU) per mouse, followed 48 hours later by injection of 5 U HCG (sigma, cat. no. CG10-1VL). Following the HCG injection, set up mating pairs consisting of one superovulated female and one male per cage. Check the mating female mice for the presence of vaginal plugs at the next morning. 25ng/ul TALEN-coding mRNA targeted to Il2rg was microinjected into the cytoplasm of pronuclear-stage embryos. Surviving embryos were surgically implanted into the oviducts of surrogate ICR female mice. The offspring were analyzed for knocking-out of Il2rg gene. All mice were bred and maintained in specific pathogen-free (SPF)-grade cages and provided with autoclaved food and water. At the end of the experiments, all animals were sacrificed according to institutional guidelines.

All experimental protocols were performed in accordance with instruction guidelines from the China Council on Animal Care and approved by the guidelines of the Ethics Committee of Animal Experiments at GIBH.

**B-ALL Patients samples**

All primary samples were obtained with informed consent for research purposes, and the procedures were approved by the Research Ethics Board of GIBH. BM and/or PB samples were obtained from 25 B-ALL patients at the time of their initial diagnosis, which was based on cell morphology (FAB classification), cytochemistry, and routine immunophenotyping[3]. All B-ALL samples were collected at Nanfang Hospital, with patients having provided informed consent and for research purposes only. All related procedures were monitored and approved by the Institutional Review Boards of Southern Medical University. Mononuclear cells were separated by density gradient centrifugation (Lymphoprep, StemCellTechnologies, Vancouver, BC, Canada). Samples were cryopreserved in liquid nitrogen in RPMI 1640 with 40% FCS and 10% dimethylsulfoxide (DMSO) or were transplanted into immunodeficient mice through retro-orbital injection. Such methods were carried out in accordance with the approved guidelines. Leukemic cells were obtained by centrifugation in density gradients and were washed twice in phosphate buffered saline (PBS). The clinical characteristics of the 25 patients are provided in Table S1 and S2.

**Xenotransplantation animal model**

Freshly isolated or thawed primary B-ALL cells (1-5×10^6^) were injected via the retro-orbital route into 8-10 weeks NSI mice. Mice were firstly given 1 Gy total body irradiation. For serial transplantations, leukemic cells of xenografts were harvested and transplanted into NSI recipients. In single-cell transplantation assay, single cells from the third xenografts were isolated by serial dilution and were individually injected into 6-8 weeks NSI mice.

**Cell culture**

All cells were incubated at 37°C in a humidified atmosphere containing 95% air and 5%CO2. OP9 stromal cells (CRL-2749,ATCC, Manassas, VA, USA) were cultured in α-MEM (HyClone, Thermo Scientific, Waltham. MAUSA) supplemented with 20% fetal bovine serum (FBS) (Gibco, Thermo Scientific, Waltham. MAUSA), 2mM L-glutamine, 100 U/ml penicillin, and 100 μg/ml streptomycin. NIH-3T3 (CRL-1658, ATCC) was maintained in Dulbecco's Modified Eagle **Medium** (HyClone, Thermo Scientific) supplemented with 10% fetal bovine serum (FBS, Gibco, Life Technologies), 2 mM L-glutamine, 100 U/ml penicillin, and 100 μg/ml streptomycin. B-ALL cells harvested from xenografts were enriched via magnetic cell sorting using anti-human CD45 MicroBeads (Miltenyi Biotec, Germany) and were cultured at an density of 1×10^6^ /ml in IMDM (HyClone, Thermo Scientific), 10% FBS (Gibco, Life Technologies), 2mM L-glutamine 100 U/ml penicillin, and 100 μg/ml streptomycin.

Before co-culture of B-ALL cells with OP9 stromal cells, OP9 cells were pre-seeded in 24-well plates and washed twice with leukemic cell culture media. For long-term expansion in vitro, the B-ALL cells should be passaged when OP9 cells nearly grow confluent. For transwell culture (Millipore, Darmstadt, Germany), B-ALL cells were cultured in the inserts, and OP9 cells were cultured at the bottom of the well, separated by a transwell. For single B-ALL cell proliferation assay in vitro, OP9 cells were seeded in 96-well plates 24 hours before seeding of individual B-ALL cells. B-ALL cells were serially diluted in 200μl culture media and were confirmed as single cells under a microscope (Axiovert A1, ZEISS, Oberkochen, Germany).

**RNA-Seq analysis**

The RNA was extracted with an RNeasy RNA Mini Kit (Qiagen, Stockach, Germany). The quality of the RNA was analyzed with an Agilent Bioanalyzer 2100 (Agilent, Santa Clara, CA, USA) before the next procedures. Poly-A library preparation and sequencing were performed according to the manufacturer’s protocols (Illumina, San Diego, CA, USA). The prepared libraries were sequenced with an Illumina GAIIx analyzer at up to 40M reads per sample, which generated 1×75 bp single-end reads.

The sequencing reads were mapped to the human RefSeq-RNA reference sequence by applying the FANSe 2 algorithm with the parameters −L85 −E3 −U0 −S10[4]. Alternative splice variants were merged[5]. Genes with at least 10 mapped reads were considered reliably detected genes[6]. These genes were further quantified on the basis of count values, which were raw counts of sequencing reads. The count values were imported into the DESeq software package[7] to calculate the up-/down-regulation of genes among samples.

**Histological analysis**

Organ or tissues samples were fixed in 10% formalin, embedded in paraffin, sectioned at 4-μm thickness, and stained with hematoxylin and eosin. Images were obtained on a microscope (Leica DMI6000B, Leica Microsystems, Wetzlar, Germany).

**Flow cytometry and cell sorting**

All antibodies used in this study are listed in Table S7. Flow cytometric analysis was performed with a FACSAria II cell sorter (BD Biosciences, San Jose, CA, USA). Approximately 1×10^6^ cells were stained with **fluorescein-**conjugated monoclonal antibodies against human leukocytes-surface antigens for 30 min at 4^o^C. PB, spleen, and BM samples from the mice were processed according to standard procedures. For cell sorting experiments, 1×10^7^ cells per sample were stained with anti-human CD45, anti-human CD34, and anti-human CD38 (eBioscience, San Diego, CA, USA). Sorted cells were reanalyzed by flow cytometry, and the purity of subpopulations was 97.3%±0.89 (n=12, Figure S4). FACS data were analyzed with FlowJo software (FLOWJO, LLC., Ashland, OR, USA).

**qRT-PCR**

Total RNA was isolated from cells with the use of TRIzol reagent (Invitrogen, Thermo Scientific, Waltham. MA USA). cDNA was obtained with the SuperScript reverse transcriptase kit (TransGene, Beijing, China) and the use of oligo-dT primers. All primers were synthesized by Invitrogen (Guangzhou, China). Gene expression was measured with the CFX96 Real-time PCR System (Bio-Rad, Hercules, CA, USA) using the SYBR Select Master Mix (Invitrogen, Thermo Scientific, Waltham. MA USA) according to the manufacturer’s protocol. All of the primers used in this study are listed in Table S8.

**Whole-exome sequencing**

Genomic DNA was extracted from indicated specimens using the DNeasy Blood & Tissue Kit (Qiagen, 69506) according to the manufacturer’s protocols. The concentration and quantity of total DNA were assessed by measuring absorbance with NanoDrop® ND-1000 and runing 0.8% Argose gel. 6ug of DNA was sheared and subject to whole-exome sequecing using the Agilent SureSelect All Exon capture probe set and sequenced by HiSeq2500. A median 7.9 Gb of unique sequence was generated for each sample. Sequence reads were aligned to the human reference genome buid 27, using Novoalign (novocraft.com).

**Statistics analysis**

Data are presented as mean ± SEM and were analyzed by IBM SPSS 20 with Student’s t test. Pearson’s Chi-square test was used to determine correlation between clinical risks or treatment outcomes and engraftments in NSI mice. Differences were considered statistically significant at p < 0.05.

1. Ye W, Jiang Z, Li GX, Xiao Y, Lin S, Lai Y, Wang S, Li B, Jia B, Li Y *et al*: **Quantitative evaluation of the immunodeficiency of a mouse strain by tumor engraftments**. *Journal of hematology & oncology* 2015, **8**:59.

2. Xiao Y, Jiang Z, Li Y, Ye W, Jia B, Zhang M, Xu Y, Wu D, Lai L, Chen Y *et al*: **ANGPTL7 regulates the expansion and repopulation of human hematopoietic stem and progenitor cells**. *Haematologica* 2015, **100**(5):585-594.

3. Coustan-Smith E, Sancho J, Hancock ML, Boyett JM, Behm FG, Raimondi SC, Sandlund JT, Rivera GK, Rubnitz JE, Ribeiro RC *et al*: **Clinical importance of minimal residual disease in childhood acute lymphoblastic leukemia**. *Blood* 2000, **96**(8):2691-2696.

4. Zhang G, Fedyunin I, Kirchner S, Xiao C, Valleriani A, Ignatova Z: **FANSe: an accurate algorithm for quantitative mapping of large scale sequencing reads**. *Nucleic acids research* 2012, **40**(11):e83.

5. Wang T, Cui Y, Jin J, Guo J, Wang G, Yin X, He QY, Zhang G: **Translating mRNAs strongly correlate to proteins in a multivariate manner and their translation ratios are phenotype specific**. *Nucleic acids research* 2013, **41**(9):4743-4754.

6. Zhong J, Cui Y, Guo J, Chen Z, Yang L, He QY, Zhang G, Wang T: **Resolving chromosome-centric human proteome with translating mRNA analysis: a strategic demonstration**. *J Proteome Res* 2014, **13**(1):50-59.

7. Anders S, Huber W: **Differential expression analysis for sequence count data**. *Genome biology* 2010, **11**(10):R106.
